# Culture-independent and culture-dependent analyses of the bacterial community in the phycosphere of cyanobloom-forming *Microcystis aeruginosa*

**DOI:** 10.1038/s41598-019-56882-1

**Published:** 2019-12-31

**Authors:** Minkyung Kim, Bora Shin, Jaebok Lee, Hye Yoon Park, Woojun Park

**Affiliations:** 1 Laboratory of Molecular Environmental Microbiology, Department of Environmental Science and Ecological Engineering, Korea University, Seoul, 02841 Republic of Korea; 2 National Institute of Biological Resources, Incheon, 22689 Republic of Korea

**Keywords:** Water microbiology, Microbial ecology

## Abstract

Confocal and scanning electron microscopic observations have previously shown the strong bacterial association of *Microcystis aeruginosa* cells on their surfaces. DNA-based analyses of the associated bacterial communities were carried out using two *M. aeruginosa* strains grown in the laboratory and eight newly collected cyanobacterial bloom samples. *M. aeruginosa* was the most predominant species (66–100%) within the phylum Cyanobacteria. *Rhizobium, Hydrogenophaga* and *Brevundimonas* species were commonly found, and *Flavobacterium* species were present in all the cyanobacterial bloom samples. In total, 396 colonies from various samples were screened, revealing that most culturable bacteria belonged to the class *Alphaproteobacteria* (19%) including *Rhizobium*, *Brevundimonas*, and *Porphyrobacter* species. The genetic variation among the *M. aeruginosa* strains and different habitat conditions may have led to the presence of distinct bacterial populations among the tested samples. Among all the tested seven culturable isolates, *Rhizobium* sp. MK23 showed the best growth-promotion effect on the axenic *M. aeruginosa* strains. H_2_O_2_ was observed to be produced during the growth of *M. aeruginosa* PCC7806 under light conditions, this strain was more resistant to H_2_O_2_ when associated with *Rhizobium* sp. MK23. Our data suggested that *Rhizobium* species along with other associated bacteria might help the growth of *M. aeruginosa* by decomposing H_2_O_2_ under the aerobic growing conditions.

## Introduction

Cyanobacterial blooms are common phenomena in several freshwater environments, including drinking water sources, around the world. These blooms are often associated with production of cyanotoxins, oxygen depletion, unpleasant odor, and ecosystem health issues^1^. The bloom-forming cyanobacteria such as *Microcystis*, *Anabaena*, and *Oscillatoria* species decrease the esthetic and recreational values of the water bodies and cause a serious threat to the ecosystems^2^. Cyanobacterial blooms are caused by high nutrient loading, elevated water temperature and radiation levels, global warming due to high CO_2_ concentration, drought, and increased water salinity^2,3^. The factors that influence cyanobacterial growth are water temperature above 25 °C, weak water current^2^, sufficient phosphorus supply, and low nitrogen/phosphorous (N/P) ratio^3^. The appearance of floating cyanobacterial blooms has been one of the most unpleasant symptoms of eutrophication^4^. Previous studies have emphasized on phosphorous as the main nutrient source affecting phytoplankton mass and eutrophication in the majority of lakes^5^. Representative bloom-forming cyanobacterial genera include *Microcystis, Anabaena*, and *Cylindrospermopsis* from freshwater sources, and *Nodularia* and *Aphanizomenon* from estuaries^6^. The *Microcystis* species, such as *M. aeruginosa, M. flosaquae, M. ichthyoblabe, M. wesenbergii*, and *M. pherta* are responsible for causing almost 90% of the cyanobacterial blooms in freshwater bodies. Of these, *M. aeruginosa* is the most commonly observed to cause cyanobacterial blooms^2,7,8^.

The colonies of *Microcystis* genus are generally about 4–5 µm in size; however, coagulated colonies range from 52–200 µm in size^9^, consequently exhibits thick aggregations of cells under natural environmental conditions^10^. *Microcystis* species are widely known to produce anatoxin-a, a neurotoxin, and microcystin which are the primary toxins found in freshwaters worldwide^11^. Microcystin synthetase gene clusters (*mcy*) comprise genes coding for polyketide synthase, peptide synthase, and mixed polyketide/peptide synthase^12^. Microcystin concentrations have been directly or indirectly determined using protein phosphatase inhibition assays, enzyme-linked immunosorbent assay (ELISA), chemical derivatization with gas chromatography– mass spectrometry analysis, and high-performance liquid chromatography coupled to either ultra-violet, photodiode array detection or mass spectrometry detection^13^. PCR amplification of the *mcy* genes has proven to be effective in distinguishing between the microcystin-producing hepatotoxic and non-toxic *Microcystis* strains^14^. For identifying *Microcystis* species in environmental samples, the most common PCR targets employed are the microcystin synthetase gene operon and *Microcystis*-specific 16S rRNA and phycocyanin (*cpcBA*) genes^15,16^, in addition to the genes related to nutrient transport and metabolism^17^. The size of *Microcystis* genomes range from 4.26 Mbp (*M. aeruginosa* PCC 9806) to 5.84 Mbp (*M. aeruginosa* NIES 843) in size; however, only 27 draft or closed genomes are available, and ≥12,000 predicted genes remain uncharacterized^18^. Moreover, since there is a large genetic variation among *Microcystis* species, the lack of species-specific genetic information complicated PCR-based differentiation of individual species^17^.

*Microcystis* species grow into large mucilaginous aggregates, which comprise a microscale mucus region called the phycosphere, and this region is generally colonized by associated bacteria^19^. Fluctuations in the bacterial diversity associated with *Microcystis-*blooms are often observed in many environmental samples^20^. *Microcystis*-associated bacteria can directly adhere to the cells on the surface of a *Microcystis* colony^21^ or colonize within the enclosed region^22^. Many associated bacteria can enhance or suppress the growth of cyanobacteria^23^, or even kill them^24^. Difficulty in obtaining the axenic cultures of *Microcystis* species is probably due to the lack of knowledge about the roles of associated bacteria in xenic cultures^25^. The photosynthetic performances and growth rates of *Microcystis* species in xenic culture are higher than those in the axenic cultures^26^. Nevertheless, the effects of bacterial association on *Microcystis* species and their underlying mechanism remain unclear. Interactions between algae and bacteria have been intensively studied (but not within phycosphere); for example, the *Emiliania huxleyi*-*Roseobacter* interaction in the marine ecosystem^27^. *Roseobacter* species promote algal growth for a short period by supplying the algae with vitamin B_12_ and phytohormones, such as abscisic acid, auxin, and gibberellins and by providing antimicrobial protection against the other bacteria^27^. In addition, the ammonium-excreting bacterium *Azotobacter vinelandii* has been found to promote microalgal growth^28^. Mutualism has been observed between *Chlorella vulgaris* and *Pseudomonas* species under photoautotrophic condition^29^.

This study examined the bacterial species that are associated with the phycospheres of 10 bloom-forming ten *Microcystis aeruginosa* (two strains were laboratory-grown and eight srains were recently collected from various cyanobacterial-bloom-forming areas in August, 2018). Culture-independent and -dependent studies were conducted in order to explore the diversity of *Microsytis*-associated bacteria. Our data here demonstrated that many *Alphaproteobacteria* including *Rhizobium* species, are the dominant bacteria in the phycosphere of *M. aeruginosa*. *Rhizobium* species along with other associated bacteria likely protect *M. aeruginosa* against oxidative stress under aerobic growth conditions and *M. aeruginosa* produces acetate for associated bacteria.

## Results

### Cyanobacterial bloom related to variation in the water characteristics

Water quality at each sampling site was assessed in order to understand the cyanobacterial bloom conditions in the environment (Fig. S1). The temperature ranged from 31.0 °C [at Daecheong lake (DC) sample] to 33.0 °C [at Hapcheon Changnyeong barrage (HC) sample] among the sample locations (Table 1, Fig. S1). The pH value ranged from 7.0 to 9.4, and the most alkaline condition was found in the DC sample. Higher alkaline conditions, such as pH 9.5, have been shown to promote the growth of cyanobacteria^30^. The dissolved oxygen (DO) levels in the DC sample was low (2.0 ± 0.22 mg/L); similarly, the other samples also presented with low DO levels (<1 mg/L). It was difficult to judge the correlation between the chlorophyll-*a* (chl-*a*) concentration and organic contents because the chemical oxygen demand (COD) values of our environmental samples were slightly lower (9.6–9.82 mg/L) than the previously reported the COD values (10.5–17.5 mg/L) of bloom-containing water samples^31^. The concentration of total nitrogen (TN) in the HC sample was 13.84 mg/L, and this level was significantly higher than those in the other sites. Additionally, HC sample also had the highest total phosphorus (TP) concentration (0.38 mg/L). The concentration of chl-*a* increased with TN and TP values, except for the Juksan barrage (JS) sample differed (Fig. S2), and indicated the severity of the cyanobacterial bloom at the corresponding sampling site (Fig. S3). With 1111.04 mg/m^3^, HC sample had the maximum chl-*a* concentration. The cyanobacteria in samples collected from Murwang reservoir (MW), Wangsong reservoir (WS), Gangjeong-Goryeong barrage (GJ), and Bohyun mountain Dam (BH) had formed small coagulates although these sites had cyanobacterial blooms. Hence, the amount of chl-*a* per volume was lower (below 10 mg/m^3^) than expected because of variation of chl-*a* concentration at different sampling points. Although the main cause of a cyanobacterial bloom could not be identified from our bloom-water characteristics, the cyanobacterial blooms and high concentrations of chl-*a* and cyanobacterial blooms in JS and HC samples were correlated with elevated TN and TP concentrations. However, regarding DC sample, the main cyanobacterial bloom was likely correlated with the high pH rather than TN or TP concentration.

**Table 1:** Measurement of the Temperature (°C), pH, DO (mg/L), Salinity (ppt), COD (mg/L), Total N (mg/L), and Total P (mg/L) of water quality of the environmental samples for determining the water quality; MW (Murwang reservoir), DC (Daecheong lake), BJ (Baekje barrage), HC (Hapcheon-Changnyeong barrage), WS (Wangsong reservoir), JS (Juksan barrage), and GJ (Gangjeong-Goryeong barrage).

Location	Temperature	pH	DO	Salinity	COD	Total N	Total P
MW	32.35 ± 1.04	7.04 ± 0.13	0.43 ± 0.06	0.13 ± 0.005	9.631	ND	0.122
WS	31.90 ± 0.67	8.40 ± 0.36	1.01 ± 0.18	0.21 ± 0.000	9.783	1.62	0.121
DC	31.00 ± 0.14	9.44 ± 0.14	1.95 ± 0.22	0.08 ± 0.000	9.774	1.97	0.082
BJ	32.67 ± 0.23	7.83 ± 0.17	0.89 ± 0.50	0.18 ± 0.000	9.599	1.83	0.078
JS	32.28 ± 0.08	7.47 ± 0.18	0.56 ± 0.19	0.15 ± 0.000	9.659	2.13	0.239
HC	32.97 ± 0.14	7.89 ± 0.11	0.51 ± 0.20	0.14 ± 0.000	9.653	13.84	0.38
GJ	31.22 ± 0.46	7.52 ± 0.26	0.74 ± 0.59	0.36 ± 0.005	9.820	ND	0.024

ND: not detected.

### Cyanobacterial coexistence with bacteria

**Figure 1:**
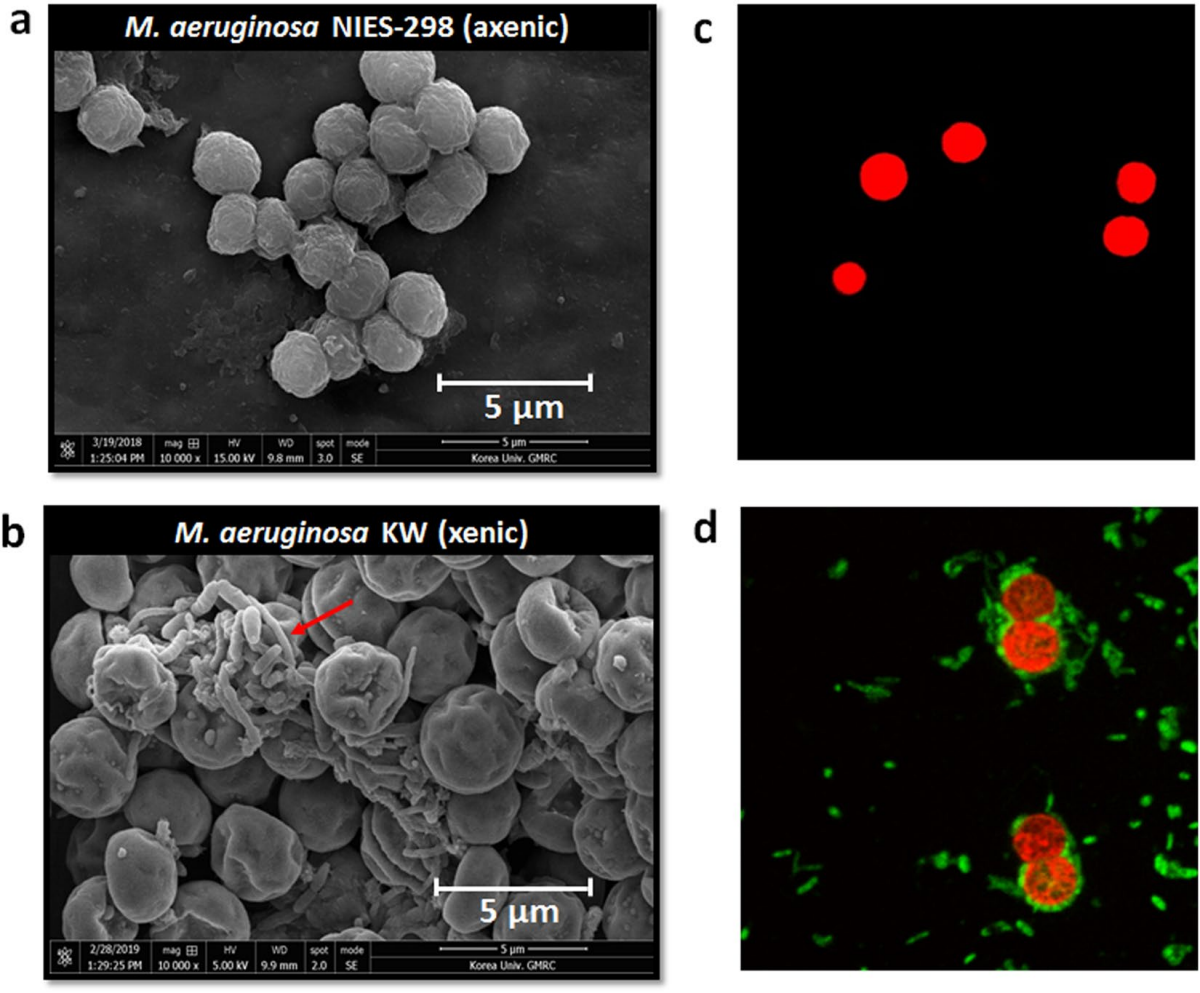
*Microcystis*-bacteria co-existence. Scanning electron microscopy (SEM) image of the (**a**) axenic *M. aeruginosa* NIES-298 culture, and (**b**) xenic *M. aeruginosa* KW. SYTO^TM^ 9 staining analysis of the (**c**) axenic *M. aeruginosa* NIES-298 and (**d**) xenic *M. aeruginosa* KW. The red and green represents *M. aeruginosa* and bacteria, respectively. The bacteria associated with the *M. aeruginosa* surface.

To test whether *M. aeruginosa*-associated bacteria were attached to *M. aeruginosa* cells, an *M. aeruginosa* KW sample was examined by field emission scanning electron microscopy (FE-SEM) and confocal laser scanning microscopy (CLSM). The cells of *M. aeruginosa* KW coexisted with various bacterium species unlike compared to the axenic *M. aeruginosa* NIES-298 (Fig. 1a,b). Some bacterium cells were found attached to the surface of *M. aeruginosa* KW. However, the bacteria were not attached to all the cells of *M.aeruginosa*, but rather clustered intensively. CLSM following SYTO 9 staining also confirmed the presence of epiphytic bacteria on the surface of *M. aeruginosa* KW cells (Fig. 1c,d). Notably, the bacteria were observed to be surrounding the *M. aeruginosa* KW cells.

### Bacterial communities in cyanobacterial phycospheres

**Figure 2:**
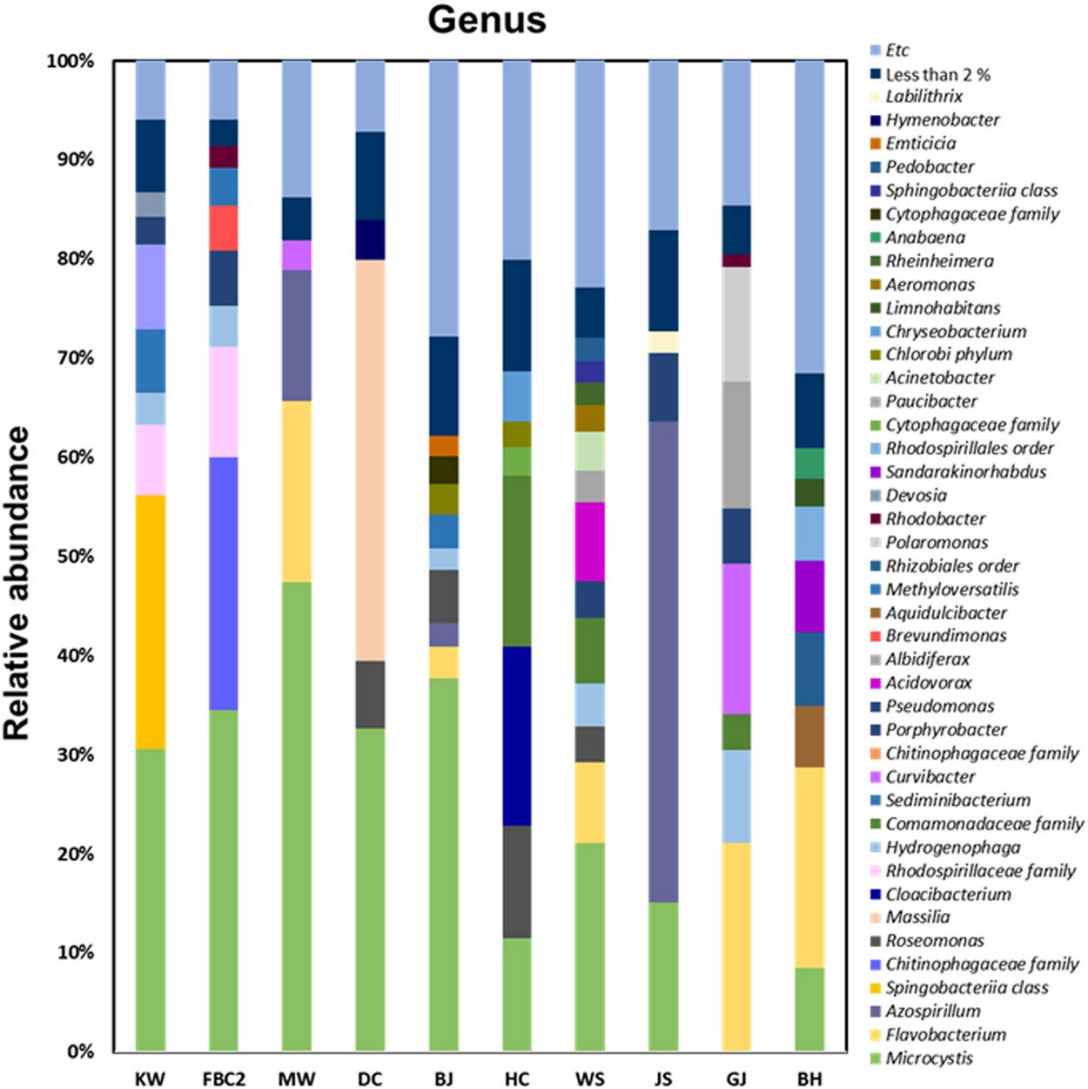
Bacterial community analysis of all the following *M. aeruginosa* samples: *M. aeruginosa* KW (KW), *M. aeruginosa* FBC000002 (FBC2), Murwang (MW), Daecheong (DC), Baekje (BJ), Hapcheon (HC), Wangsong (WS), Juksan (JS), Gangjeong (GJ), and Bohyun (BH). The genera with <1% abundance were included as well. The laboratory-cultured samples consisted of simple bacterial communities, whereas the environmental samples had various bacterial populations.

Bacterial communities of two laboratory grown samples (KW and FBC2) and eight environmental samples were analyzed by microbiome taxonomic profiling of the epiphytic bacteria isolated using 3-μm pore filters from the cyanobacterium samples. Differential interference contrast microscopy results showed that *Microcystis* species and various phytoplanktons were present together in the environmental samples (Fig. S4). *M. aeruginosa* was the most predominant species (66–100%) within the phylum cyanobacteria (Fig. 2). At genus level, *Flavobacterium* species were represented in all the environmental samples and were a dominant species in the MW, WS, GJ, and BH samples (10–35%; Fig. 2); however, it was absent from the laboratory samples. Approximately 22 species (except *Flavobacterium* species) were exclusively present in the environmental samples but not abundantly (<8%), and 11 species, including *Rhizobium, Brevundimonas, Porphyrobacter, Hydrogenophaga*, and *Sediminibacterium* were present in all the samples (Fig. S5). Two laboratory *M. aeruginosa* strains were dominated by different unclassified bacteria of the class Sphingobacteriia (KW: 36.86% and FBC2: 38.9%). Moreover, the predominant species in KW sample also existed in the JS sample, and the primary species of FBC2 was present in all the environmental samples, except for DC and MW samples. Unclassified *Chitinophagaeceae* species, the second dominant species in KW sample (12.37%), were present in all the samples, except for DC sample. Interestingly, at genus level, the bacterial community of the DC sample revealed that genus *Massilia* (40.45%) was more abundant than *M. aeruginosa* (32.66%) at the genus level. *M. aeruginosa* KW and *M. aeruginosa* FBC2 were similar in bacterial community to each other and simpler than the environmental samples, except for the five bacteria at the genus level (<1%). The environmental samples presented a wider variety of phyla (>15 phyla) than the laboratory samples (8 and 10 phyla in KW and FBC2, respectively). The OTUs of the environmental samples were significantly higher than those of the laboratory strains; among the environmental samples, the highest OTU value was observed in the BH sample (1380), and the lowest in the DC sample (384) (Table 2). ETC is a group of strains that contributed <1% of the total abundance of genus. The presence of ETC also revealed that the environmental samples were more heterogeneous than the laboratory samples.

**Table 2:** Results of the bar-coded pyrosequencing and diversity indices of two laboratory strains and eight environmental samples.

Sample	Reads per sample	Average length (bp)	OTUs	Number of species found	Chao1	Shannon
KW	138,693	412.6	350	99	350.55	2.44
FBC2	135,777	410.1	342	110	343.70	2.23
MW	83,642	408.8	655	373	670.05	2.20
DC	38,436	414.9	384	184	396.66	2.29
BJ	64,915	410.7	1306	570	1320.99	3.83
HC	63,341	415.8	815	324	822.97	3.51
WS	80,158	418.0	949	674	974.88	4.28
JS	74,589	409.3	1060	445	1068.14	2.84
GJ	59,841	422.3	1174	613	1194.81	4.21
BH	72,252	411.0	1380	629	1394.12	4.43

### Different genotypes in *Microcystis aeruginosa* strains

In our growth experiments, which were performed using a PhotoBiobox, the optimum light and temperature conditions were different for four cyanobacterial bloom samples (Fig. S6). Interestingly the growth of all the strains was affected by temperature rather than light intensity, and optimal growth was observed at <25 °C rather than 30 °C. The 16S rRNA gene sequencing revealed a high similarity of >99% among the *Microcystis* species in all the samples, and this observation suggested that the 16S rRNA analysis alone was not enough to distinguish the genotypes of *M. aeruginosa* strains. Nevertheless, a few sequence differences were observed among the sequences. The 16S rRNA gene sequencing revealed the difference of T vs. C at nucleotide 223, and T vs. G at nucleotides 235 and 236 (Fig. S7). The result of PCR analysis, targeting the microcystin gene cluster (*mcyA, mcyB*, and *mcyC*), confirmed the presence of toxic *Microcystis* species in our samples. An electropherogram of the PCR products for *mcyA* revealed that MW, DC, BJ, JS, and BH samples contained the *mcyA* gene (Fig. S8a). HC and GJ samples did not contain the *mcyB* gene, whereas the *mcyC* gene was detected in all the environmental samples. The 16S rRNA gene sequencing indicated that GJ and BH samples contained the same species, but the two species differed in *mcy-*gene-targeting PCR analysis (Fig. S8). Interestingly, the species in the MW and BJ samples have the *mcyA, mcyB*, and *mcyC* genes, but their microcystin-LR concentrations were <0.1 µg/L (Fig. S8b). This data suggested that these samples had *M. aeruginosa* strains producing structurally different microcystins that could not be detected with the kits used in this study. HC sample did not generate a product with the conventional microcytin PCR primer set^32–34^, but produced microcystin-LR, indicating the complexity of the genotypes and evolution of *M. aeruginosa* strains. Our data suggested that genotypic differences among the *M. aeruginosa* strains may contribute to the variations in associated bacterial community structures.

### Diversity structure and phylogenetic analyses of the culturable bacteria

**Figure 3:**
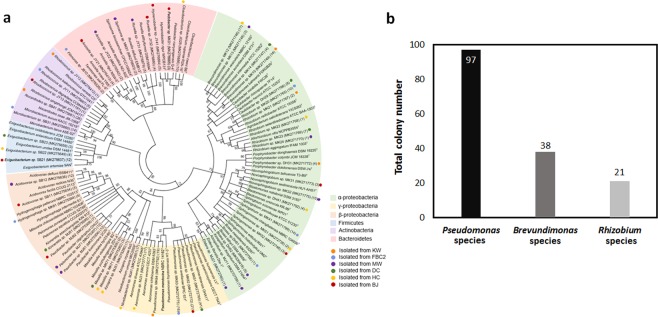
Culture-dependent analysis of the 16S rRNA gene sequences. (**a**) Neighbor‐joining phylogenetic tree of the isolated bacteria. Each color represents a phylum level of the bacterial community. The associated bacteria were isolated from KW, FBC2, MW, DC, BJ and HC samples. The number in parentheses indicates the number of isolated associated bacteria. Most bacteria belonged to the class *Alpha-proteobacteria*. GenBank accession numbers are provided in parentheses. (**b**) *Pseudomonas* species were the most frequently isolated species from the culturable bacteria.

To characterize the culturable bacteria, 396 colonies were isolated from six samples including the laboratory samples (KW, FBC2, MW, DC, BJ, and HC), indicating the presence of high percentage of *Microcystis* species in the bacterial community. Although the chl-*a* concentration in the MW sample was low (6.29 mg/m^3^), this sample was selected for culture-dependent analysis because it had 100% *M. aeruginosa* presence as the phylum cyanobacteria. Three types of culture media used for bacterial growth did not reveal any significant difference in colony formation. The strains with identical BOX-PCR patterns were used to perform the amplification of 16S rRNA genes (Fig. S9). A phylogenetic analysis was performed using the identified gene sequences and closely related type strains from the EzTaxon Database (Fig. 3a). *Rhizobium* species were commonly found in five samples, except for the BJ sample (Fig. 3b); however, it was present in all the samples from the culture-independent analysis. *Pseudomonas* species were abundantly found in KW, FBC2, DC, and BJ samples, and *Aeromonas* species were dominant in HC sample. With respect to culturable bacteria in MW sample, *Acidovorax, Pelomonas*, and *Paucibacter* species were found in similar proportions. Various bacterial species were isolated (12 genera) from the environmental MW sample, however, only six bacterial species were identified in the laboratory-grown KW sample. Interestingly, one-third of all the culturable genera belonged to the *Alphaproteobacteria* class. These results indicate that culture analysis and DNA-based bacterial community analysis resulted in different diversity structures probably because of different bacterial growth properties.

### Effect of associated bacteria on the growth of *M. aeruginosa* cells

**Figure 4:**
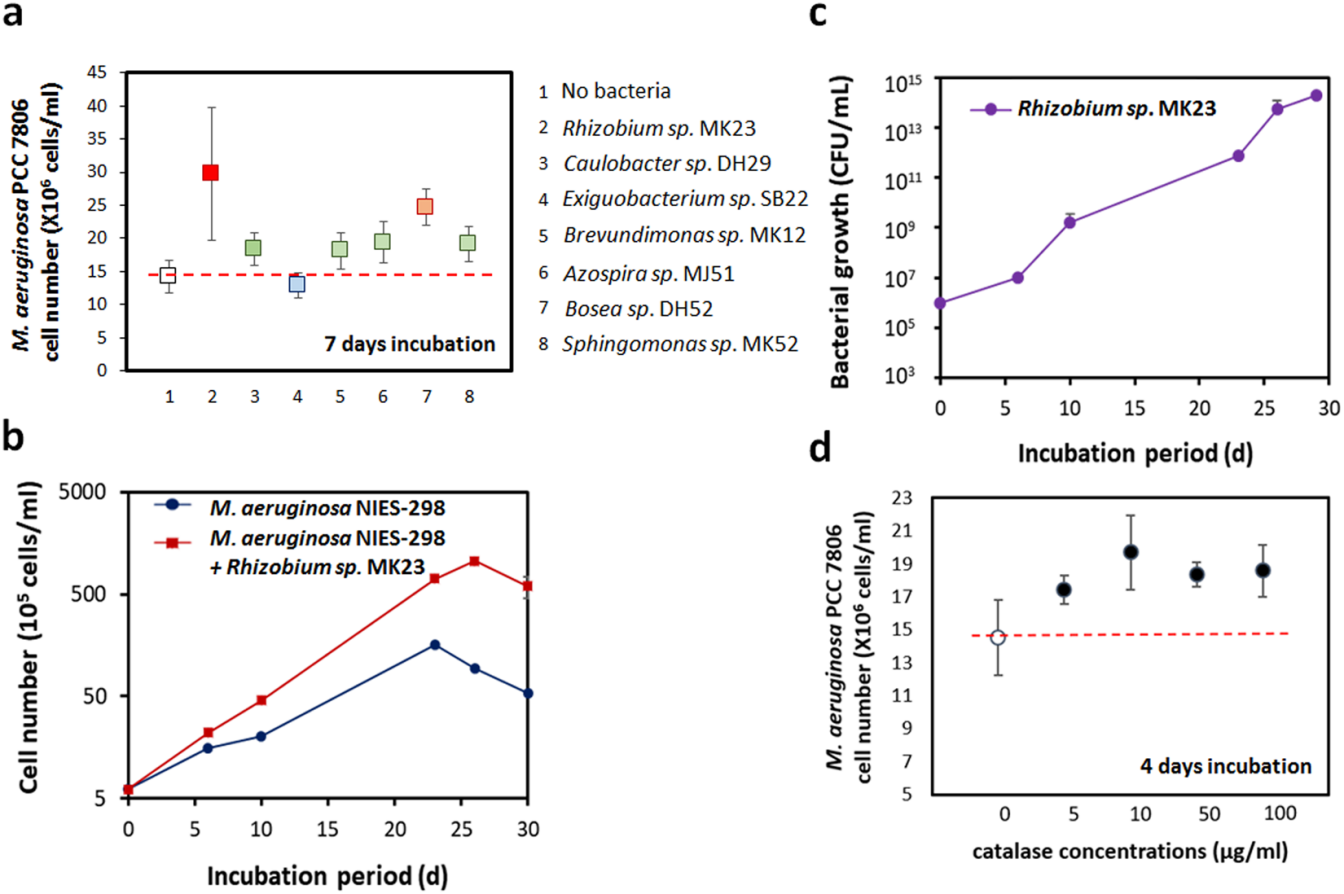
(**a**) Co-culture experiments of *M. aeruginosa* PCC7806 with seven genera, *Rhizobium sp*. MK23 apparently promoted the growth of *M. aeruginosa* PCC7806 for 7 days. **p < 0.05. (**b**) *M. aeruginosa* NIES-298 grew faster with *Rhizobium* sp. MK23. The *M. aeruginosa* NIES-298 culture and co-culture started degrading on 23 and 26, repectively. (**c**) *Rhizobium sp*. MK23 in co-culture grew at a density of 10^14^ cells/ml in 30 days. (**d**) Addition of catalase promoted the growth of *M. aeruginosa* PCC7806 for 4 days.

**Figure 5:**
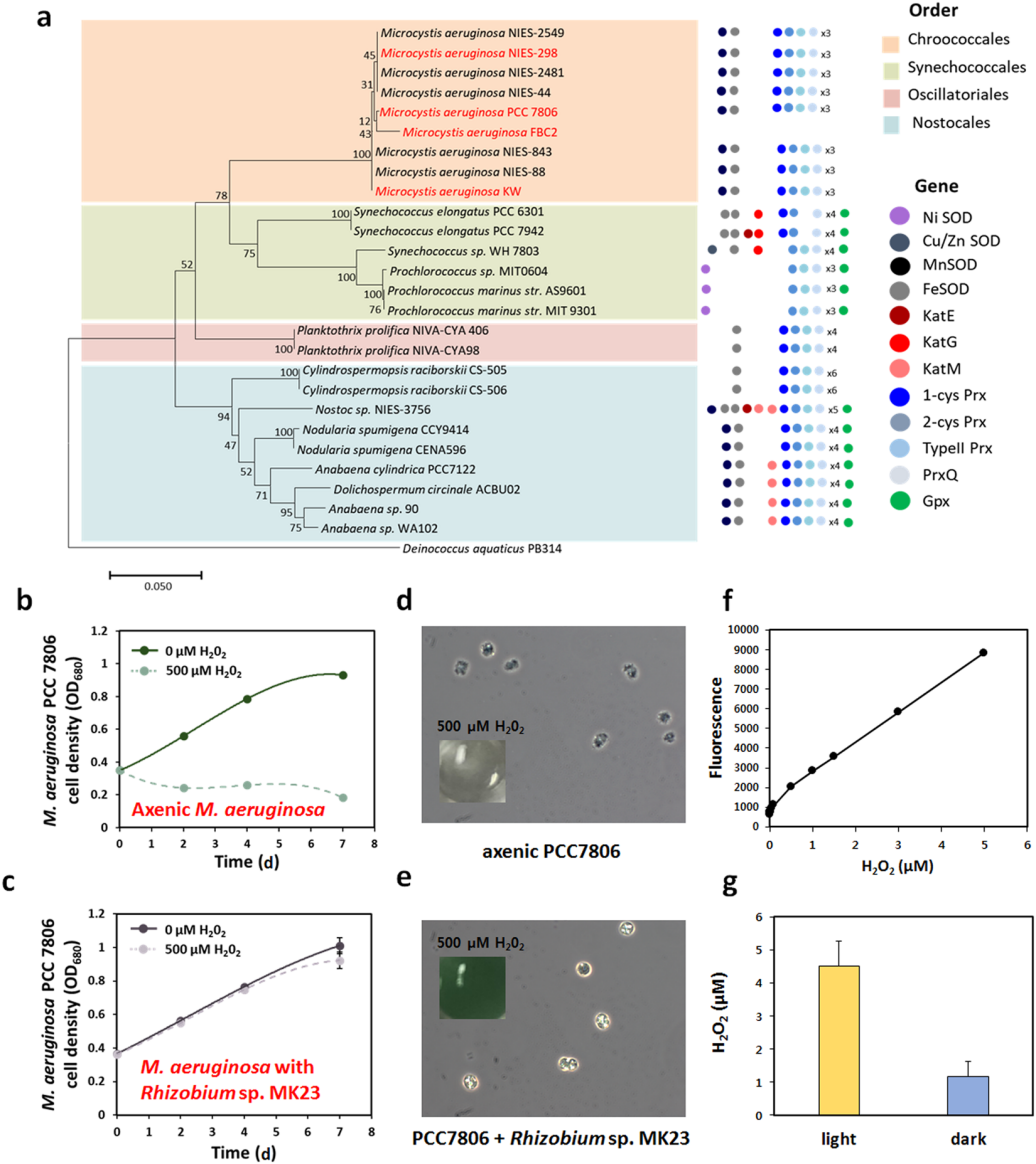
*M. aeruginosa* cells are sensitive to oxidative stress. (**a**) The listed cyanobacterial antioxidant genes are SOD, superoxide dismutase; Kat, catalase; Prx, peroxidase; and Gpx, glutathione peroxidase. *M. aeruginosa* does not have a gene for producing a catalase. (**b**) Axenic of *M. aeruginosa* PCC7806 and *M. aeruginosa* PCC7806 with *Rhizobium* sp. MK23 were treated with 500 µM concentration of H_2_O_2._ After 7 days of treatment, the growth of *M. aeruginosa* PCC7806 was retarded under H_2_O_2_. (**c**) The growth of axenic *M. aeruginosa* PCC7806 with *Rhizobium* sp. MK23 did not change significantly. (**d**) The axenic PCC7806 treated with H_2_O_2_ was transparent in color and had an unusual cell shape. (**e**) PCC7806 co-cultured with *Rhizobium* sp. MK23 were still green and had a normal, round shape. (**f**) The standard curve for H_2_0_2_ concentrations in H_2_O_2_ assay. (**g)** In the H_2_O_2_ assay, the concentrations of H_2_O_2_ were much higher in the light condition than in dark.

Seven cultured genera were randomly selected, and co-culture experiments were performed with axenic *M. aeruginosa* PCC7806. All the tested bacteria except *Exiguobacterium* species had positive effects on the growth of *M. aeruginosa* PCC7806 compared with the growth of axenic PCC7806 (Fig. 4a). Among the seven genera we tested, *Rhizobium* and *Bosea species* appeared to be the best *M. aeruginosa*’ growth-promoting bacteria for *M. aeruginosa* (Fig. 4a). *Rhizobium* species were present with different degrees in all the samples, and *Bosea* species were present in all the samples, except for WS sample. *Rhizobium* sp. MK23 also promoted the growth of the axenic *M. aeruginosa* NIES-298 strain (Fig. 4b). The maximum density of axenic *M. aeruginosa* was 1.6 × 10^7^ cells/ml on day 23; however, that of the xenic culture (incubated with *Rhizobium sp*. MK23) was 1.1 × 10^8^ cells/ml on day 26. Interestingly, the cell number of *M. aeruginosa* NIES-298 started to decreases on the 23^rd^ day, whereas the xenic culture began to decreases on the 26^th^ day. Furthermore, *Rhizobium* sp. MK23 continued to grow, with cell population increasing from 1 × 10^6^ cells/ml to 2 × 10^14^ cells/ ml within 30 days (Fig. 4c). The addition of catalase resulted in the enhanced growth of the axenic *M. aeruginosa* PCC7806 (Fig. 4d). Our genome analysis indicated that *M. aeruginosa* strain does not have a gene for producing catalase (Fig. 5a). The sensitivity of *M. aeruginosa* PCC7806 with *Rhizobium* sp. MK23 to H_2_O_2_ (500 µM) was much lesser than that of the axenic *M. aeruginosa* PCC7806 (Fig. 5b,c). The axenic PCC7806 treated with H_2_O_2_ observed an unusual cell shape. However, PCC7806 co-cultured with *Rhizobium* sp. MK23 had still a normal, round shape (Fig. 5d,e). Under the light condition, the concentration of H_2_O_2_ was 4.5 µM in the axenic culture of axenic *M. aeruginosa* PCC7806, and the concentration of H_2_O_2_ was decreased under the dark condition (Fig. 5f,g). Our data strongly suggested that associated bacteria protect *M. aeruginosa* cells under oxidative stress, leading to better growth of the xenic culture.

## Discussion

Cyanobacterial blooms of *Microcystis* species are characterized by the formation of large colonies through collision after cell division and proliferation^35^. Flocculated colonies float with buoyancy and form a layer on the surface of the water, gradually covering the entire water surface^36^. At the sampling sites, cyanobacterial blooms were found as dense layers of cells on the water surfaces or as sparsely floating coagulates. Owing to the differences in the timing of sampling, the concentrations of chl-*a* widely differed among the sites. Previous studies have reported significant differences among the communities of free-living and cyanobacteria-associated bacteria as a result of several weeks of blooming^36^. Therefore, bacterial communities and nutrient qualities may change depending on the cyanobacterial bloom periods.

The major factors that promote the growth of cyanobacterial biomass include high concentration of nutrients such as phosphorus and nitrogen^37^. Bacterial communities, as well as the levels of water nutrients, are affected by the surrounding area was near a farmland or livestock farms, the concentrations of TN and TP were relatively higher, suggesting that nutrients present in water are related to the surrounding environment. Globally, approximately 50% of the N fertilizer applied to cultivation systems is not absorbed by plants and is released into the environment as ammonia (NH_3_), nitrate (NO^3−^), and nitrous oxide (N_2_O)^38^. Many of our sampling areas were surrounded by farmlands except the DC sample, which was collected from a drinking water reservoir. Therefore, the number of OTUs in DC sample was not significantly lower than those in other environmental samples (Table 2). The *Rhizobium* and *Azospirillum* species were the predominant bacteria in the JS sample. These species have the nitrogen-fixing ability, which might help the growth of *M. aeruginosa*^39,40^. WS sample had the lowest concentration of total nitrogen, which may be related to the presence of a second dominant bacterium, a nitrate-reducing *Acidovorax* species^41^. The growth of *Microcystis* species in natural environments can be affected by not only abiotic factors but also biotic facters involved in the N and P cycles of associated bacteria.

The culture-independent analysis results revealed that the two laboratory strains had lower levels of ETC (<1%) than the environmental samples, indicating that there is less species richness in domesticated samples (Fig. 2). Our data suggested that only important bacteria interacting with *Microcystis* species remain as cyanobacterial bloom samples continued growing in the laboratory. The culture-independent analysis results revealed that some bacteria were common to all the samples, but the dominant species were different. Different optimal temperature and light conditions suggested that four samples had genotypically different *M. aeruginosa* species (Fig. S6). An electropherogram of PCR products for the microcystin gene was likely to miss the presence of genes because of primer mis-matches. It is difficult to distinguish the diversity of *Microcystis* species only by microcystin gene PCR or 16S rRNA gene sequencing because of the significant genetic variations among *Microcystis* strains^17^. A previous study has shown that seventeen various strains of *M. aeruginosa* different from *M. aeruginosa* NIES-843 had >99% 16S rRNA sequence identity^10^. Therefore, some associated bacteria may be specifically to certain *Microcystis* species.

The genus *Polynucleobacter*, which is the most well-known genus predominantly found in freshwater habitats, can be regarded as a good indicator of water quality. *Polynucleobacter* species accounted for <1% of all the species, indicating poor water qualities of our environmental samples. Actinobacteria are also known to be abundant in freshwater, but their number is decreased in nutrient-rich ecosystems and is inversely proportional to cyanobacteria^42–44^. The culture-independent analysis results showed that the proportion of Actinobacteria was also <1%. *Flavobacterium* species was predominant in four samples and abundantly present within the cyanobacterial aggregates^45^. This species may be crucial for degrading the cyanobacterial hepatotoxin^46^. The bacterial community structure determined by the culture-dependent analysis was different from that of the DNA-based bacterial community analysis. According to the culture-dependent analysis results, *Pseudomonas* species was the most prevalent in the DC sample but accounted for only 0.14% according to the results of the DNA-based bacterial community analysis. *Pseudomonas, Brevundimonas* and *Rhizobium* species were the most abundant according to the results of the culture-dependent analysis, by which 396 colonies were screened from six samples (Fig. 3). However, *Brevundimonas* species, but not *Pseudomonas* strains, were estimated to be more abundant in many samples by DNA-based analysis.

**Figure 6:**
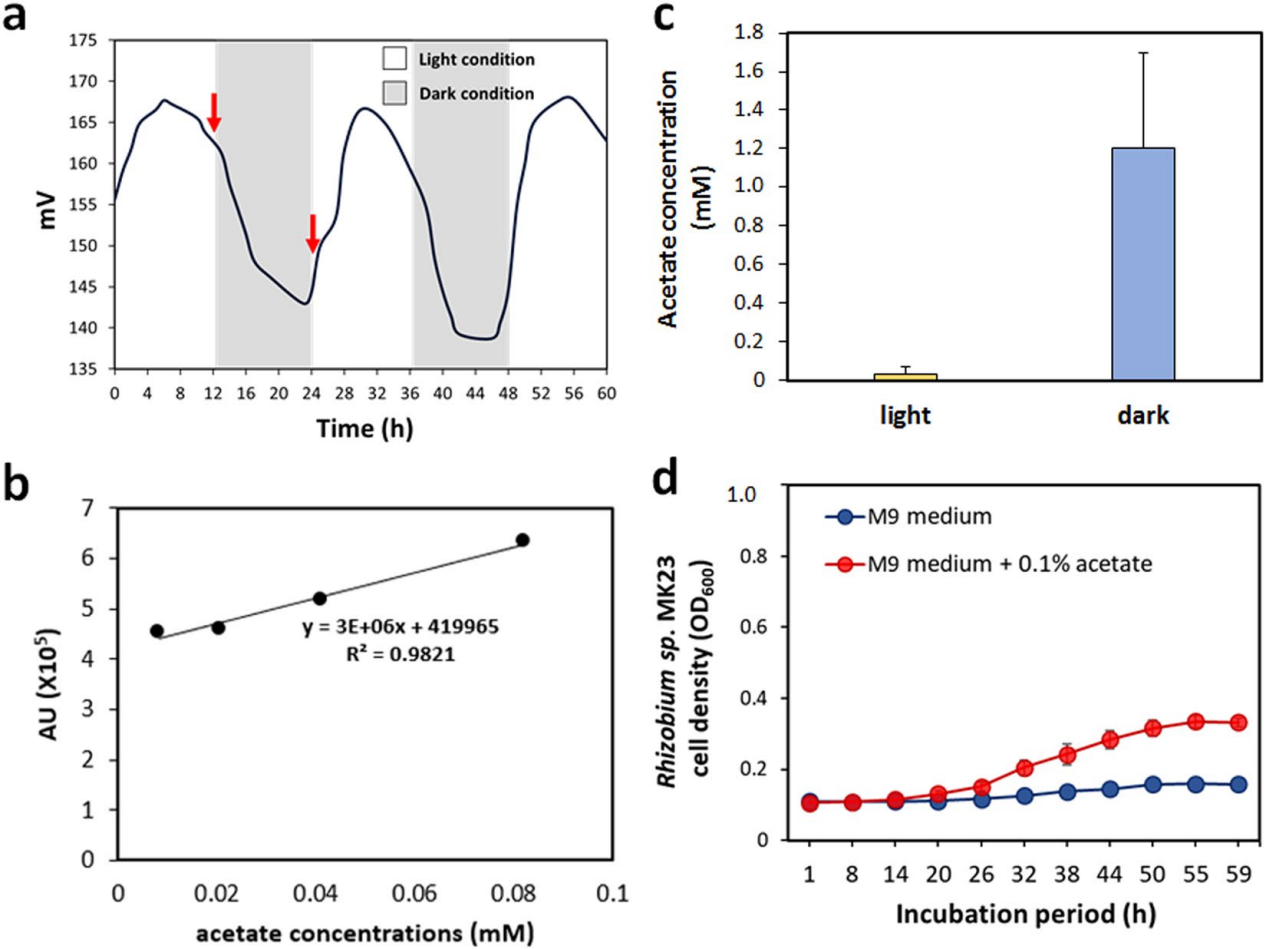
(**a**) Dissolved oxygen (DO) changes during the growth of *M. aeruginosa* PCC7806 under the light and dark conditions for 60 h. In *M. aeruginosa* PCC7806 cultures, DO increased in the light condition and decreased in the dark condition. Cell supernatant was extracted at 12 h and 24 h (red arrows). (**b**) The standard curve for the acetate concentrations in HPLC analysis. (**c**) The HPLC analysis results showed that the concentration of acetate was much higher in the dark condition than in the light. (d) Growth curve of *Rhizobium sp*. MK23 under 0.1% acetate.

The growth of axenic *M. aeruginosa* PCC7806 with several isolates including *Rhizobium* species was boosted for 7 days-culture experimement (Fig. 4a). The *Rhizobium* species can do nitrogen fixation and produce catalases^47^. However, *Microcystis* species lack the genes for catalase^48^ and nitrogenase. Our catalase test showed that *Rhizobium* sp. MK23 has catalase activity (Fig. S10a). The cell number of the axenic PCC7806 in BG11 without nitrogen source was more decreased than that of the PCC7806 with *Rhizobium* sp. MK23 (Fig. S10b). We speculated that *Rhizobium* species help the growth of *M. aeruginosa* by providing catalase function and fixed nitrogen resources. Indeed, the addition of catalase improved the axenic *M. aeruginosa* culture growth, which partly supporting our speculation (Fig. 4d). Under the light conditions, the concentration of H_2_O_2_ in *M. aeruginosa* PCC7806 culture was 4.5 µM, and the *M. aeruginosa* PCC7806 with *Rhizobium* sp. MK23 was less sensitive to H_2_O_2_ than axenic PCC7806 (Fig. 5). DO changes in axenic *M. aeruginosa* PCC7806 culture under the light and dark conditions were measured for 60 h (Fig. 6a). Under the light condition, O_2_ was produced during photosynthesis (Fig. 6a). *M. aeruginosa* PCC7806 is known to ferment endogenously stored glycogen to ethanol, acetate, CO_2_, and H_2_ under the dark and anaerobic conditions^49^. The acetate level in axenic *M. aeruginosa* PCC7806 culture increased under the dark conditions (Fig. 6b,c). *Rhizobium sp*. MK23 could use acetate as a carbon source (Fig. 6d). Hydrogen-oxidizing *Hydrogenophaga* species was present in all the samples although it occupied a minor proportion. Our *M. aeruginosa* species might produce H_2_ gas as a fermentation product, which can be a good substrate for *Hydrogenophaga* species. More experiments seem necessary to prove this hypothesis. Taken together, our data suggested that different associated bacteria were identified in the phycospheres of *M. aeruginosa* strains probably due to genetic and physiological heterogeneities among these strains. Although further analysis is needed to have a full understanding of their mutual interaction, we demonstrated that metabolites, including acetate, and catalase activities could be shared by two different microorganisms.

## Methods

### Cyanobacterial strains and culture conditions

Experiments were performed *in vitro* using *M. aeruginosa* KW, *M. aeruginosa* FBC000002 (*M. aeruginosa* FBC2), and eight collected cyanobloom samples. *M. aeruginosa* KW was isolated from Wangsong reservoir, Republic of Korea by researchers from the Korea Research Institute of Bioscience & Biotechnology. *M. aeruginosa* FBC2 was isolated from the Nakdong River, Republic of Korea and cultured by researchers from Freshwater Bioresources Culture Collection. Two axenic strains were used in the experiments to prevent strain-specific bias. The axenic culture of *M. aeruginosa* NIES-298 was established by the National Institute for Environmental Studies, Japan, and the axenic culture of *M. aeruginosa* PCC7806 was established by the Pasteur Culture collection of Cyanobacteria, France. These strains were cultured in BG11 medium and at 25 °C and a light intensity of 50 µmol m^−2^s^−1^ with a photoperiod of 12 h light/12 h dark under controlled conditions in a growth chamber

### Experimental sites and water sampling

Cyanobacteria samples were collected from the following eight sites of cyanobacterial blooms in the Republic of Korea: Murwang reservoir (MW; 37°22′46.5′′N, 126°49′59.5′′E), Wangsong reservoir (WS; 37°18′47.0′′N, 126°56′53.8′′E), Daecheong lake (DC; 36°21′21.8′′N, 127°33′30.4′′E), Baekje barrage (BJ; 36°16′30.5′′N, 126°53′27.9′′E), Juksan barrage (JS; 34°58′29.9′′N 126°36′37.0′′E), Hapcheon Changnyeong barrage (HC; 35°35′32.5′′N, 128°21′25.4′′E), Gangjeong Goryeong barrage (GJ; 35°48′43.0′′N 128°28′38.9′′E), and Bohyun mountain dam (BH; 36°07′25.4′′N 128°56′38.0′′E) in August of 2018 (Fig. S1). The samples were collected from surface water and the water source was profiled *in situ* at 1 m depth for temperature, pH, DO, and salinity using a multiparameter water quality meter (Table 1) (YSI 556, Yellow Springs Instruments, USA). Regarding BH sample, it was impossible to measure the water quality. In addition, the samples were analyzed for chemical oxygen demand (COD_Mn_) and total nitrogen (TN), and total phosphorus (TP) concentrations using HS-CODMn-L, HS-TN-L (CA), and HS-TP-L kits (Humas, Republic of Korea). Chl-*a* was extracted with 90% acetone using a spectrophotometer (Spark, Germany). Each water sample was mixed with 1 L of water was added in a plastic bottle and preserved at 4 °C while transporting to the laboratory.

### Confocal and scanning electron microscopy

*M. aeruginosa* and epiphytic bacterial co-cultures were examined via confocal and scanning electron microscopes (SEM). *M. aeruginosa* KW was cultured in BG11; 1 ml of suspension was added to 5.9 µl of SYTO 9 green-fluorescent nucleic acid stain and vortexed for 30 s. *M. aeruginosa* cells were stained, for 5 min in the dark. SEM observation was also performed to confirm that various bacteria were attached to the cells of *M. aeruginosa*. To visualize the coexisting condition, 1 ml of cells was harvested via centrifugation (1 min at 11,500 rcf) and gently washed twice with PBS. The cells were primarily fixed at 4 °C for 4 h (Karnovsky’s fixation method). Thereafter, they were washed thrice at 4 °C with 0.05 M potassium phosphate buffer for 10 min each at 4 °C. The cells were additionally fixed with a mixture of 0.1 M potassium phosphate buffer and 2% osmium tetroxide at 4 °C for 2 h. Next, they were washed twice with distilled water at room temperature. The cells were dehydrated using increasing ethanol concentrations (30, 50, 70, 80, and 90%, and then treated thrice with 100% ethanol, for 10 min each) at room temperature. The samples were coated with platinum prior to examination by FE-SEM (FEI, Japan).

### Analysis of bacterial communities in the *M. aeruginosa* phycospheres

Each sample was filtered through a 3-μm (47 mm, Macherey-Nagel, Germany) pore size polytetrafluoroethylene filter paper and washed twice to remove the free-living bacteria. Metagenomic DNA was extracted from microorganisms in the water using a FastDNA Spin Kit (MP Biomedicals, USA) according to the manufacturer’s instructions, and the DNA yield was quantified using a NanoDrop spectrophotometer (BioTek, USA). The V3-V4 hypervariable region of 16S rRNA gene from the genomic DNA was amplified using primers 341 F (5′-TCGTCGGCAGCGTC-AGATGTGTATAAGAGACAG-CCTACGGGNGGCWGCAG-3′) and 805 R (5′-GTCTCGTGGGCTCGG-AGATGTGTATAAGAGACAG-GACTACHVGGGTATCTAATCC-3′). The amplified products were confirmed by agarose gel electrophoresis. The amplicons were purified by Agencourt AMPure XP (Beckman Coulter, Republic of Korea) and quantified using a Quanti-iT Picogreen dsDNA Assay kit. Equimolar concentrations of each amplicon from the different samples were pooled and purified using Agencourt AMPure XP (Beckman Coulter, Republic of Korea). All sequencing procedures were conducted by ChunLab (Republic of Korea). The sequences obtained were compared and classified using the EzTaxon Database (http://www.ezbiocloud.net). The operational taxonomic units (OTUs) among the samples were obtained with a taxonomic composition using the CLcommunity program (ChunLab, Republic of Korea). The raw sequences obtained from each sample were deposited in the GenBank SRA archive and are available with the following accession numbers: BioProject accession number PRJNA528487; Samples accession numbers: SRX5561067~SRX5561076.

### Comparative analysis of the *Microcystis* strains

The similarity in 16S rRNA sequence among the strains was analyzed using MegAlign software. Percentage identity showed the difference in *M. aeruginosa* sequence among the environmental samples, based on the KW strains. To compare the optimal culture conditions in BG11 medium for each strain, screening of cyanobacteria was performed with a PhotoBiobox^50^ at a temperature of 15–40 °C and light intensity of 20–160 µmol m^−2^ s^−1^ with a photoperiod of 10 h light/10 h dark. All the strains were incubated in 96-well plates with a working volume of 200 µL. After cultivation for 3 days, fluorescence was measured with the excitation at 420 nm and emission detection at 680 nm using a spectrophotometer (Spark, Germany). Three pairs of primers targeting the *mcyA, mcyB*, and *mcyC* genes were used for the amplification (Table 3)^32–34^. After separating the aggregated colonies by a pipette, the cells were sonicated. A total volume of 20 µL containing 10 µl of cells, 2 µl of each primer, 1.6 µl of dNTPs, 2 µL of 10 × buffer, 0.2 µL of Han-taq (Genenmed, Republic of Korea) polymerase, and 10 µL ddH_2_O was used for each PCR amplification. The following PCR protocol was used: 94 °C for 5 min (1 cycle); 94 °C for 1 min, 60 °C for 30 sec (*mcy*A) or 52 °C for 30 sec (*mcy*B and *mcy*C), and 72 °C for 1 min (34 cycles), and 72 °C for 10 min (1 cycle). The amplificons were analyzed by agarose (0.8%) gel electrophoresis. The microcystin-LR concentrations in the environmental sample were measured using an ELISA kit (Abnova, Taiwan),.

**Table 3:** Primers used in the study.

Gene region and primer	Sequence (5′-3′)
**Microcystis 16S rRNA**	
209F	ATGTGCCGCGAGGTAAACCTAAT
409R	TTACAATCCAAAGACCTTCCTCCC
***mcyA***	
MSF	ATCCAGCAGTTGAGCAAGC
MSR	TGCAGATAACTCCGCAGTTG
***mcyB***	
2156F	ATCACTTCAATCTAACGACT
3111R	AGTTGCTGCTGTAAGAAA
***mcyC***	
PSCF1	GCAACATCCCAAGAGCAAAG
PSCR1	CCGACAACATCACAAAGGC

### Isolation of culturable bacteria from *M. aeruginosa*

Experiments were conducted after filtering the samples using a 3-μm (47 mm, Macherey-Nagel, Germany) pore size polytetrafluoroethylene filter paper, followed by washing. To obtain various bacteria, the culturabilities of the bacteria were investigated by using R2A medium, R2A medium at 1/10 of its normal concentrations, or nutrient agar medium at 1/100 of its normal concentrations as the growth medium^51^. The colonies were isolated after 4 days of incubation at 30 °C, and at the end of microcosm incubation, 66 colonies per sample were streaked on a fresh medium. Approximately 34 colonies were selected in R2A medium, with 16 colonies in 1/10 R2A, and 1/100 NB mediums each. Prior to bacterial identification, BOX PCR was performed to determine the presence of the same bacteria. The BOX primer^52^ was inserted in a 20 μl reaction volume that contained 10 μl of cells, 2 μl of 10× taq buffer, 4 μl of primer (5 pmol; BOXA1R, 5′-CTA CGG CAA GGC GAC GCT GAC G-3′), 1.6 μl of dNTPs (10 mM of each), and 0.2 μl of Han-taq (Genenmed, Republic of Korea) polymerase. The reaction mixture was amplified using a Mastercycler nexus X2 (Eppendorf, Germany) with the PCR conditions as follows: an initial denaturation step at 95 °C for 2 min (1 cycle); 94 °C for 3 s, 92 °C for 30 s, 50 °C for 1 min, and 65 °C for 8 min (34 cycle), with a final extension at 65 °C for 8 min (1 cycle). The PCR amplification products (10 μl aliquots) were analyzed by 0.8% agarose gels electrophoresis in Tris-acetate EDTA (TAE) buffer (Bioneer, Republic of Korea). Subsequently, the gels were stained with ethidium bromide (EtBr, 5 μl), and were visualized under UV light.

### Phylogenetic analysis of isolated bacteria

In this study, culturable bacteria were identified by partial sequencing of 16S rRNA genes. PCR amplification and sequencing of the 16S rRNA gene from the isolates were performed using universal primers (27 F, 5′-AGAGTTTGATCMTGGCTCAG-3′ and 1492 R, 5′-GGTTACCTTGTTACGACTT-3′). The PCR was performed with 2 µl of each primer, 1.6 µl of dNTPs, 2 µL of 10 × buffer, and 0.2 µL of Han-taq (Genenmed, Republic of Korea) polymerase. The following PCR protocol was used: 94 °C for 90 s (1 cycle); 94 °C for 45 s, 50 °C for 45 s, and 72 °C for 45 s (40 cycles) and 72 °C for 5 min (1 cycle). Pyrosequencing of the PCR product was performed by Macrogen (Republic of Korea). The obtained sequences were compared and classified using the EzTaxon Database (http://www.ezbiocloud.net). Similar sequences were grouped into OTUs based on the manual comparison. The 16S rRNA gene sequences of each sample of the culturable bacteria were aligned using MEGA 7.0 software. After trimming the unaligned regions, a neighbor-joining phylogenetic tree was constructed using MEGA software. The 16S rRNA sequences of the culturable bacteria have been deposited in the National Center for Biotechnology Information (NCBI) GenBank.

### Co-culture experiment of *M. aeruginosa* with culturable bacteria

The bacteria used in the experiment were selected from those that were commonly detected or occupied a large proportion in all the samples and those that were present in only 1–2 samples. *M. aeruginosa* PCC7806 culture in BG11 media was inoculated with 10^6^ cells/ml bacteria, and one of the *M. aeruginosa* PCC7806 cultures was left uninoculated. The cultures were grown with 12 h light/12 h dark cycle for 7 days at 25 °C. The growth of *M. aeruginosa* PCC7806 cells was measured by a hemocytometer. Becasue the *Rhizobium* species was the best growth- stimulating bacteria for *M. aeruginosa* PCC7806, co-culture experiments with *M. aeruginosa* NIES-298 and *Rhizobium* species were intensively conducted for 30 days. The bovine liver catalase (20,658 units/ml enzyme; Sigma-Aldrich, USA) was added to axenic *M. aeruginosa* PCC7806 culture for 4 days, and the culture growth was monitored by a hemocytometer. Catalase activity depends on the conversion of the oxidation state cobalt (II) to cobalt (III) by H_2_O_2_ in the presence of biocarbonate solution as previously reported^53^. The concentration of H_2_O_2_ in the culture of *M. aeruginosa* PCC7806 was measured using the Amplex red hydrogen peroxide/peroxidase assay kit (Thermo Fisher Scientific, USA). *M. aeruginosa* PCC7806 culture in BG11 media without nitrogen source was inoculated with 10^7^ bacterial cells/ml and negative control has no *Rhizobium* sp. MK23 cells. Because the growth of *Rhizobium* sp. MK23 depends on carbon sources from PCC7806, the 1% acetate was added to BG11 without nitogen source. The DO levels in the *M. aeruginosa* PCC7806 culture under the light and dark conditions were measured using a Unisense O_2_ sensor (Aarhus, Denmark) for 60 h. The same amount of cell supernatant was extracted after 12 h in the light and after 12 h in the dark. Samples were filtered with a 0.45-µm filter and stored at −20 °C until measurement. The concentrations of acetate in the supernatants of *M. aeruginosa* PCC7806 growth media were measured by HPLC (Waters Co. USA) equipped with refractive index and photodiode array detector. The chromatography was performed on a Bio-Rad Aminex HPX-87H ion-exchange column (7.8 × 300 mm; Bio-Rad Laboratories, USA) at room temperature using 8 mM H_2_SO_4_ as the mobile phase at a flow rate of 0.1 mL/min. The injection volume was set to 15 µL, with the detection at 210 nm. The growth of *Rizobium* sp. MK23 was evaluating the OD_600_ after adding 0.1% acetate to the M9 media.

### Ethical statement

This study did not comprise any experiment or analysis performed on human participants or animals.

## Supplementary information

Supplementary Information
