# Supplementary material for: Culture-independent and culture-dependent analyses of the bacterial community in the phycosphere of cyanobloom-forming Microcystis aeruginosa
Source: Sci Rep. 2019 Dec 31;9:20416. doi: 10.1038/s41598-019-56882-1 (PMC6938486; doi:10.1038/s41598-019-56882-1)
Supplement: Supplementary file 1 — Supplementary Information [file 41598_2019_56882_MOESM1_ESM.docx]

**Culture-independent and culture-dependent analyses of the bacterial community in the phycosphere of cyanobloom-forming *Microcystis aeruginosa***

**Minkyung Kim^1^, Bora Shin^1^, Jaebok Lee^1^, Hye Yoon Park^2^, and Woojun Park^1^**

^1^Laboratory of Molecular Environmental Microbiology, Department of Environmental Science and Ecological Engineering, Korea University, Seoul 02841, Republic of Korea.

^2^National Institute of Biological Resources, Incheon 22689, Republic of Korea.

**Running title:** Bacterial community in the *Microcystis aeruginosa* phycosphere

**Keywords:** Cyanobacterial bloom, *Microcystis aeruginosa,* Microbial community, Epiphytic bacteria, Cyanobacteria-associated bacteria interaction

***Corresponding author:** Dr. Woojun Park, Department of Environmental Science and Ecological Engineering, Korea University, Seoul 02841, Republic of Korea.

**E-mail:** wpark@korea.ac.kr

**Fax:** +82-2-953-0737

**Phone:** +82-2-3290-3067


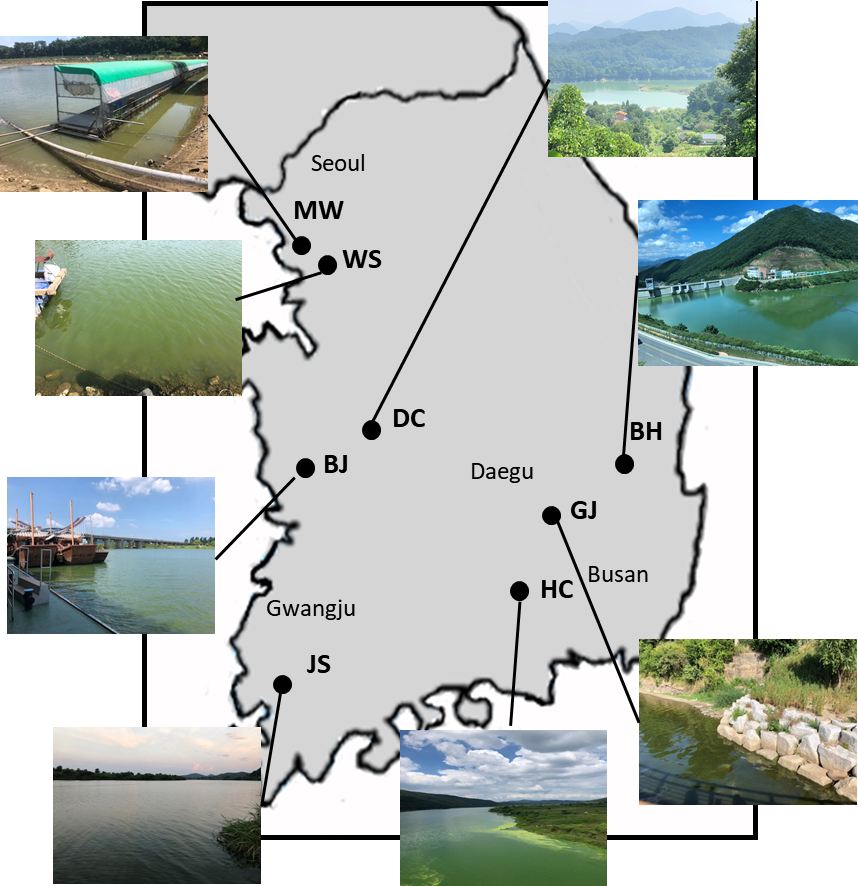


**Supplementary Fig. S1.** Cyanobacterial blooms that occurred in the Republic of Korea in August of 2018. Sampling areas: MW (Murwang reservoir), WS (Wangsong reservoir), DC (Daecheong lake), BJ (Baekje barrage), JS (Juksan barrage), HC (Hapcheon-Changnyeong barrage), GJ (Gangjeong-Goryeong barrage), and BH (Bohyun mountain Dam).


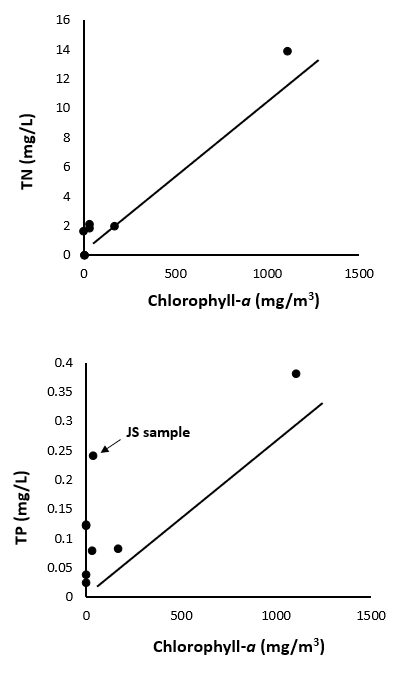


**Supplementary Fig. S2.** Correlations between chlorophyll-*a* and TN/TP concentrations. Both TN and TP cocncentrations appear to be proportional to chlorophyll-*a* concentration.


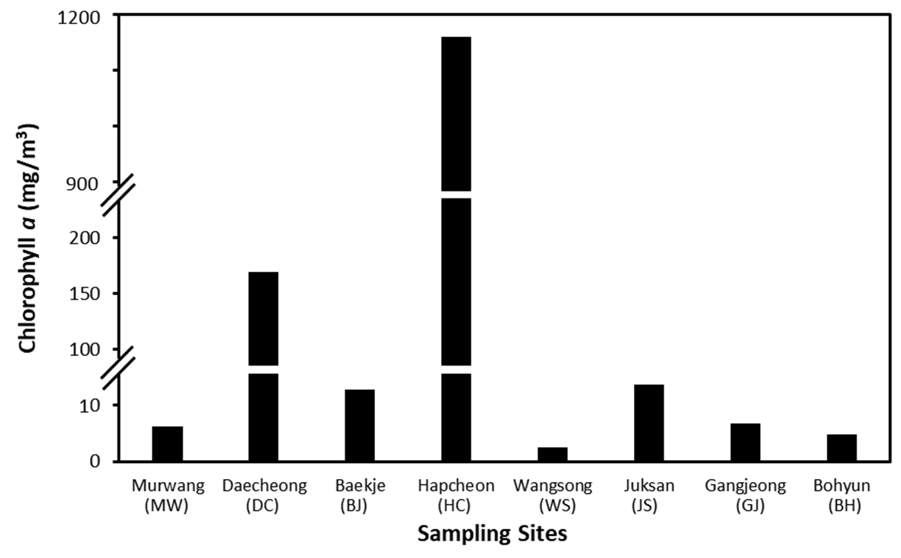


**Supplementary Fig. S3.** The chlorophyll-*a* concentrations of the environmental samples. HC sample had the highest chlorophyll-*a* concentration. HC and DC samples displayed the cyanobacterial bloom in the net form, whereas the other samples displayed the aggregated form.


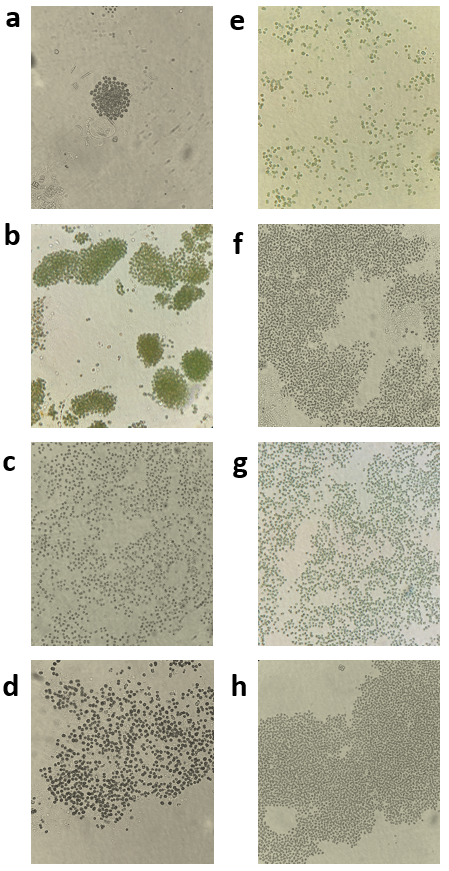


**Supplementary Fig. S4.** Differential interference contrast microscopic observation of the eight environmental samples from the following sites. (a) Murwang (MW) reservoir, (b) Daecheong (DC) lake, (c) Baekje (BJ) barrage, (d) Hapcheon-Changnyeong (HC) barrage, (e) Wangsong (WS) reservoir, (f) Juksan (JS) barrage, (g) Gangjeong (GJ)-Goryeong barrage and (h) Bohyun (BH) mountain dam. The morphologies of the *Microcystis* species in all the samples were examined.


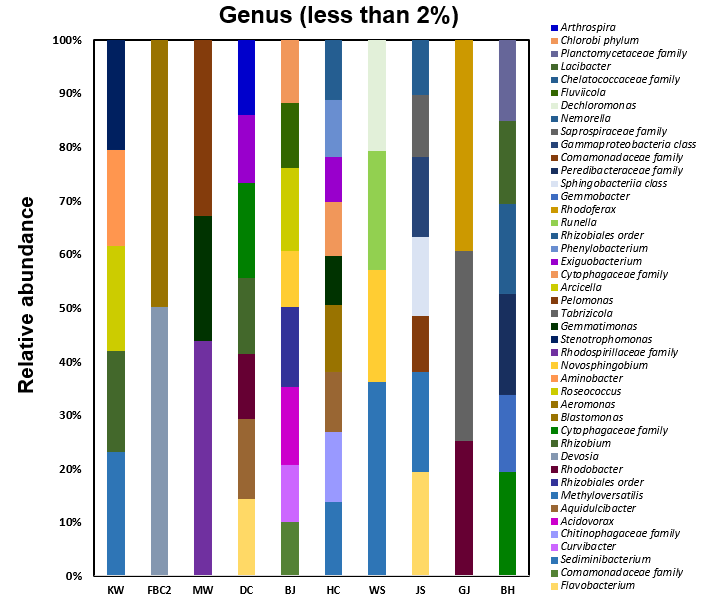


**Supplementary Fig. S5.** Culture-independent analysis **(**< 2%): *M. aeruginosa* KW (KW), *M. aeruginosa* FBC000002 (FBC2), Murwang (MW), Daecheong (DC), Baekje (BJ), Hapcheon (HC), Wangsong (WS), Juksan (JS), Gangjeong (GJ), and Bohyun (BH).


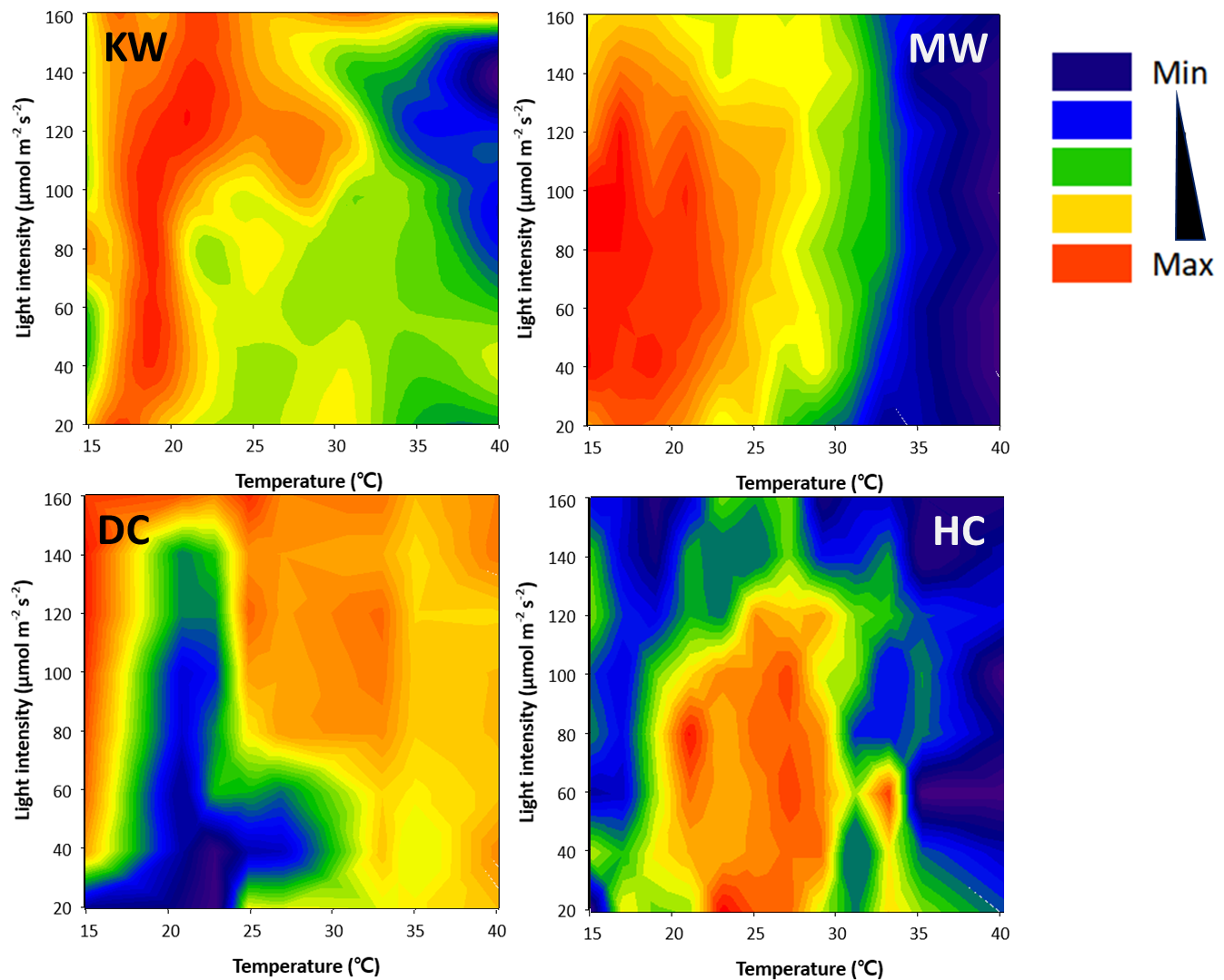


**Supplementary Fig. S6.** Screening for the optimum culture condition for each sample. The optimum temperature and light intensity for the laboratory-cultured *M. aeruginosa* KW were 20 °C and high intensity, respectively. However, the optimum conditions varied among the environmental samples varied due to the presence of other cyanobacterial species.


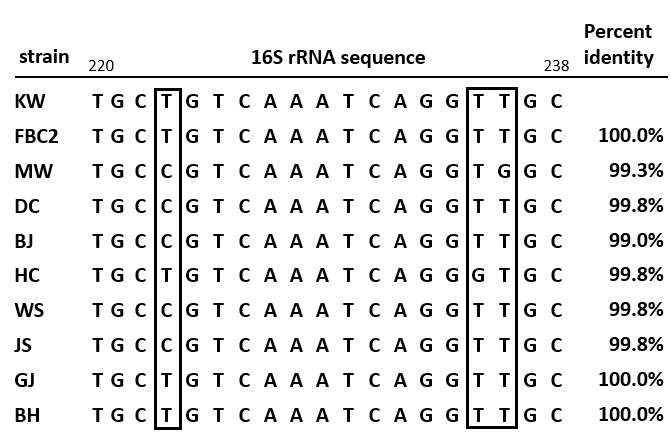


**Supplementary Fig. S7.** The differences among the *Microcystis* 16S rRNA gene sequences obtained from the samples. The percent identity is relative to the 16S rRNA gene sequences of the KW strain and indicates that although 99% of the samples exhibited high similarity (> 99%), some samples consisted of different species.


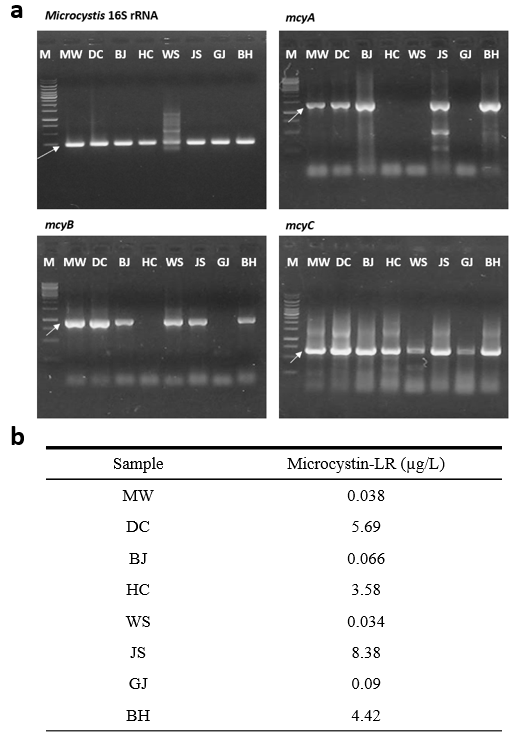


**Supplementary Fig. S8.** (a) Electropherograms of the PCR amplicons from the *mcy* gene. MW: Murwang reservoir sample, DC: Daecheong lake sample, BJ: Baekje barrage sample, HC: Hapcheon-Changnyeong barrage sample, WS: Wangsong reservoir sample, JS: Juksan barrage sample, GJ: Gangjeong-Goryeong barrage and BH: Bohyun mountain dam sample. The existence of *mcy* genes from *Microcysti*s species proves that some species are different. (b) Microcystin-LR concentrations (µg/L) determined using the an ELISA-kit.


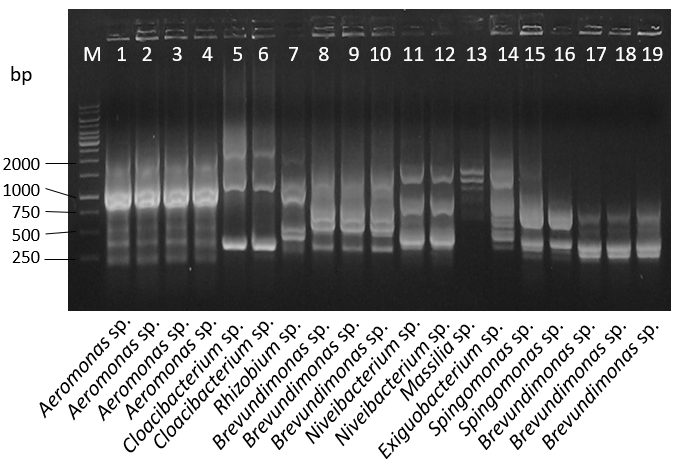
 **Supplementary Fig. S9.** BOX-PCR band patterns of bacteria isolated from the HC sample. One type of pattern indicates one species of bacteria. 1−4: *Aeromonas sp.* HC, 5−6: *Cloacibacterium sp.* HC, 7: *Rhizobium sp.* HC, 8−10: *Brevundimonas sp.* HC, 11−12: *Niveibacterium sp.* HC, 13: *Massilia sp.* HC, 14: *Exiguobacterium sp.* HC, 15−16: *Sphingomonas sp.* HC, and 17−19: *Brevundimoas sp.* HC.


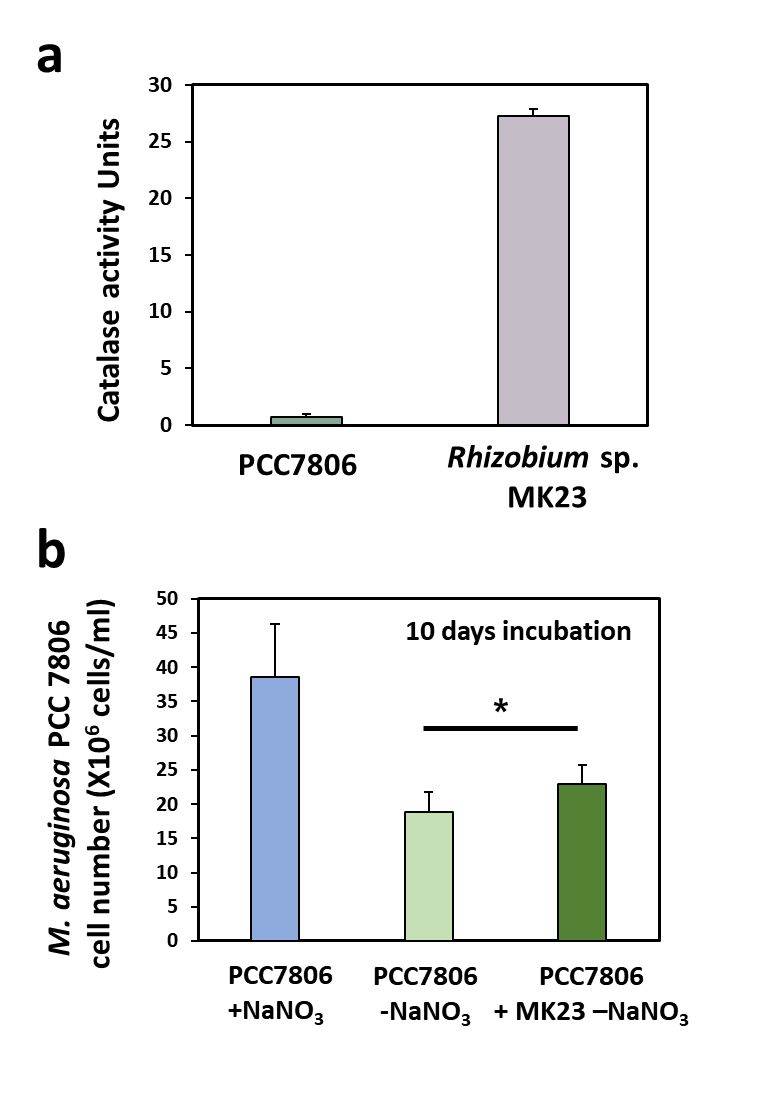


**Supplementary Fig. S10.** (a) The catalase activities of *M. aeruginosa* PCC7806 and *Rhizobium* sp. MK23. *Rhizobium* sp. MK23 was shown to exhibit higher catalase activity than *M. aeruginosa* PCC7806. (b) The growth of *M. aeruginosa* PCC7806 was defective in BG11 without nitrogen source. The cell number of the axenic PCC7806 was more decreased than that of the PCC7806 with *Rhizobium* sp. MK23. *p< 0.5.
